# Knowing who to trust: exploring the role of ‘ethical metadata’ in mediating risk of harm in collaborative genomics research in Africa

**DOI:** 10.1186/1472-6939-15-62

**Published:** 2014-08-13

**Authors:** Jantina de Vries, Thomas N Williams, Kalifa Bojang, Dominic P Kwiatkowski, Raymond Fitzpatrick, Michael Parker

**Affiliations:** 1 Department of Medicine, University of Cape Town, Anzio Road Observatory, Cape Town 7925, South Africa; 2 Kenya Medical Research Institute (KEMRI)/Wellcome Trust Programme, Centre for Geographic Medicine Research, Coast, Kilifi District Hospital, PO Box 230, Kilifi 80108, Kenya; 3 Medical Research Council Unit, PO Box 273, Banjul, The Gambia; 4 Wellcome Trust Centre for Human Genetics, University of Oxford, Oxford, UK; 5 Wellcome Trust Sanger Institute, Hinxton, UK; 6 Nuffield Department of Population Health, University of Oxford, Old Road Campus, Oxford OX3 7LF, UK; 7 The Ethox Centre, Nuffield Department of Population Health, University of Oxford, Old Road Campus, Oxford OX3 7LF, UK

**Keywords:** Data sharing, Genomics, Africa, MalariaGEN, Ethnic stigmatisation, Secondary use, Ethics, Ethnic groups

## Abstract

**Background:**

The practice of making datasets publicly available for use by the wider scientific community has become firmly integrated in genomic science. One significant gap in literature around data sharing concerns how it impacts on scientists’ ability to preserve values and ethical standards that form an essential component of scientific collaborations. We conducted a qualitative sociological study examining the potential for harm to ethnic groups, and implications of such ethical concerns for data sharing. We focused our empirical work on the MalariaGEN Consortium, one of the first international collaborative genomics research projects in Africa.

**Methods:**

We conducted a study in three MalariaGEN project sites in Kenya, the Gambia, and the United Kingdom. The study entailed analysis of project documents and 49 semi-structured interviews with fieldworkers, researchers and ethics committee members.

**Results:**

Concerns about how best to address the potential for harm to ethnic groups in MalariaGEN crystallised in discussions about the development of a data sharing policy. Particularly concerning for researchers was how best to manage the sharing of genomic data outside of the original collaboration. Within MalariaGEN, genomic data is accompanied by information about the locations of sample collection, the limitations of consent and ethics approval, and the values and relations that accompanied sample collection. For interviewees, this information and context were of important ethical value in safeguarding against harmful uses of data, but is not customarily shared with secondary data users. This challenged the ability of primary researchers to protect against harmful uses of ‘their’ data.

**Conclusion:**

We identified three protective mechanisms – trust, the existence of a shared morality, and detailed contextual understanding – which together might play an important role in preventing the use of genomic data in ways that could harm the ethnic groups included in the study. We suggest that the current practice of sharing of datasets as isolated objects rather than as embedded within a particular scientific culture, without regard for the normative context within which samples were collected, may cause ethical tensions to emerge that could have been prevented or addressed had the ‘ethical metadata’ that accompanies genomic data also been shared.

## Background

Genomic studies generate vast amounts of data that are investigated for significant associations between disease phenotype and genetic variants, following a wider transition in science from hypothesis-driven to data-driven research. Characteristic of this development is the generation of large, often publicly available datasets, the absence of specific hypotheses and the reliance on bioinformatics infrastructure to manage and analyse these. In genomics research, the need for very considerable sample numbers to allow for the generation of sufficiently large datasets has also meant that such research is increasingly collaborative in nature.

When genomics research happens in collaborations, data and samples are usually shared between collaborators. In addition to this kind of sharing, genomic researchers are also increasingly expected to share data with researchers who were not initially involved in the collaboration. The general expectation is that the utility of genomic data is greatly increased when such data are made available to the wider scientific community, and that this will reduce the costs of research whilst simultaneously speeding up the process of scientific discovery [1,2]. Furthermore, the sharing of data is expected to reduce the number of people from whom samples need to be collected afresh for research – thus reducing the possibility for adverse events.

The sharing of data raises particular ethical issues, many of which have been described in the literature. The two main challenges identified are the possibility that (knowledge about) individuals may be identified on the basis of their genetic information [3,4] and that researchers in resource-rich countries have an unfair advantage in relation to researchers in poorer settings [5]. This is particularly important when the research makes use of samples that were collected by researchers in resource-poor settings. A third challenge that has been identified relates to the possibility that the sharing of genomic data could cause stigma for the population groups involved in the study [6].

To date, data sharing in genomics has not received the kind of uptake that was anticipated [7]. One explanation given is that data sharing practices sit uncomfortably with research cultures that continue to reward publication of journal articles over the public release of data [8]. This suggests that the incentive to share is just one amongst many for genomics researchers. Another explanation is that shared data are of limited utility to other researchers [7], particularly because secondary information about how the data were generated and curated is usually omitted from the databases [9,10]. The concept of ‘metadata’ refers to such additional information that describes all the processes that lead to the generation of the data that is being shared [11-13]. Such processes include, for instance, the conditions under which samples were obtained; the criteria used to identify disease phenotypes; the process followed for DNA isolation; the methods, machines and chemicals used for amplification and sequencing; and the curation and processing of data prior to it being shared. Metadata allow data users to assess the validity of the data [14] and to place data in context. The absence of metadata can make it difficult or impossible to use the genomic data.

One significant gap in literature around data sharing concerns questions around how it impacts on scientists’ ability to maintain and preserve values and ethical standards that form an essential component of scientific collaborations. In this paper, we will describe how the recognition of the importance of preserving high ethical standards in the context of a requirement for data sharing was identified and addressed in a large genomic collaboration examining malaria, the MalariaGEN study. We also show how it was in the context of requirements for data sharing that the use of ethnicity data was identified as a practical ethical issue requiring careful consideration.

### Ethnicity in data sharing

The MalariaGEN project spans research institutions based in Africa, Asia and Europe, and is one of the few large-scale genomic studies undertaken in Africa today. Whereas genomic research has until recently tended to focus on diseases affecting people in high-income countries, genomics research tools are now increasingly used to investigate the genetic or molecular basis of complex diseases in low-income countries including those on the African continent. For a number of reasons the processing, storage and analysis of the large numbers of samples required for genomic studies in Africa tends to be located in high-income settings outside Africa. To date, whilst hundreds of Genome Wide Association (GWA) studies have taken place focussing on a wide range of conditions, hardly any of these have been applied to diseases that primarily affect people in developing countries [15,16].

One methodological challenge facing genomics research in Africa arises out of the high population diversity present there. High population diversity constitutes a methodological problem in genomic research that needs addressing because of its potential to act as a confounder in such research [17], and this problem is more pronounced when applying genomic methods to research involving African participants [18]. One solution used by researchers to address this problem is to stratify the analysis of genomic data by ethnicity to ensure that population structure can be accounted for in any analysis [19]. For this reason, the collection of genomic samples and data in such studies includes the collection of information about participants’ ethnicity.

Whilst offering important methodological benefits, the structuring of genomic analyses along ethnic or racial lines has generated considerable concern amongst social scientists and ethicists about the potential for stratified genomic research results to increase stigmatisation of or discrimination against the ethnic or racial groups included in the studies [20,21]. Controversy surrounding the use of samples from the Havasupai in the United States [22,23] offers one example to indicate the possibility that genomic research could harm the population groups included in the research.

## Methods

In this paper we draw on empirical data collected in a study described in detail elsewhere [24]. For this study, we conducted qualitative interviews with fieldworkers, researchers, ethics committee members and representatives of funding bodies in Kenya, The Gambia and the United Kingdom. In addition to the interviewing component of the study the current paper also draws on a document analysis conducted on MalariaGEN project documents. In preparing this manuscript, we have adhered to the RATS guidelines [25].

### The MalariaGEN consortium

The MalariaGEN Consortium is a network of researchers examining the genomic basis of resistance against malaria (http://www.malariagen.net), the first phase of which ran from 2005 to 2010. It incorporated approximately 50 principal investigators from Africa, Asia, Europe and the United States, 30 of whom work at 18 different research institutions in Africa. The project involved the genetic screening of samples from patients suffering from malaria, and from unaffected (healthy) children and adults.

MalariaGEN primary investigators have a variety of disciplinary backgrounds, including human genetics, paediatrics and epidemiology. It is a considerably heterogeneous group that comes together around a shared interest in using whole-genome methods to study malaria. Individuals pertaining to the MalariaGEN network met frequently over the course of 5 years – network meetings were organised on an annual basis, and a variety of other workshops and events also brought together members of the network. Human genetic samples were collected from MalariaGEN research participants in a wide range of settings in Africa, ranging from rural areas that are far removed from healthcare facilities, to more urbanised settings with referral hospitals. Samples were drawn from many different ethnic groups in Africa. DNA extraction took place at the research institutes, after which the DNA samples were exported to the UK for processing.

### Data collection and analysis

#### Document analysis

In order to map the use of ethnicity as a topic of discussion and concern in the MalariaGEN Consortium, we conducted a textual analysis of MalariaGEN project documents relating to ethnicity. A total of 54 project documents as well as personal notes of meetings were analysed. The following categories of documents were selected for this component of the study: all documents pertaining to the organisation of the MalariaGEN Consortium and the scientific studies; documents pertaining to ethical discussions, and policy documents where ethnicity was discussed. Included in the study were the minutes of meetings of the MalariaGEN programme management committee (18 documents), meeting notes of four scientific workshops (4 documents), reports and minutes of three ethics workshops (5 documents), documents regarding data release and data sharing (20 documents) and research proposals and other project policies (9 documents).

All project documents were coded manually in several rounds of coding using a thematic, progressive coding strategy [26]. Initially, all documents were read and all instances where ethnicity was discussed were highlighted. The highlighted text was then read and general topics were identified, and notes made of these. In subsequent rounds of coding, themes and sub-themes were identified in the way in which ethnicity was discussed in the Consortium. A draft of the initial analysis was circulated to selected researchers in the MalariaGEN project to seek comments and ensure the validity of our observations.

#### Interviews

We conducted 49 semi-structured interviews with various stakeholders in the MalariaGEN project. A previous article [24] described our analysis of this data in as far as it pertains to issues relating to ethnic stigmatisation. We conducted interviews with: MalariaGEN researchers (20 interviews); members of ethics committees who reviewed MalariaGEN project proposals (12 interviews); fieldworkers collecting MalariaGEN samples (15 interviews); and with members of the funding bodies that supported MalariaGEN research (2 interviews). MalariaGEN researchers were interviewed at the time of project meetings or research visits. Sixteen of the 20 MalariaGEN researchers interviewed currently work in Africa, whilst 11 of these come from African countries. One researcher works in the UK, and three others work in Asia. Interviews were conducted between June 2008 and October 2009, in the UK, The Gambia and Kenya (see De Vries et al. 2012 for more information [24]). Interviews covered: the current practice of using, defining and measuring ethnicity; awareness of particular ethical issues in using ethnicity for genomics research; issues in identifying ethnic groups and genomic data in research and publications; implications of labelling ethnic groups; issues in the sharing and re-use of ethnic data in genomics; and possible solutions to the challenges identified. Topic guides were adapted to suit the experience of the participants in the four categories. Data was analysed iteratively throughout this study, and interviews were conducted until no new issues, themes or insights were generated during the interviews or coding [27]. Interviews were recorded and transcribed verbatim. Data were analysed inductively using specialized software [28]. The first stage of open coding was followed by hierarchical coding where emerging patterns and themes in the data were established [26]. Interpretations of the data were discussed amongst the research team. Early insights were reviewed critically in subsequent rounds of coding and analysis to explore their authenticity and appropriateness. The use of detailed fieldnotes was essential in this process to trace the development of insights and understandings and offer a means for critical reflection.

### Ethics approval and consent

This study was reviewed and approved by the Oxford Tropical Research Ethics Committee in the UK (OX 22-08), the KEMRI/National Ethical Review Committee (SCC4547) and The Gambia Government/MRC Laboratories Joint Ethics Committee (SCC1137v2). All interviewees gave informed consent prior to the interview. Consent was given for participation in the study, for recording of the interview, and for the subsequent use of anonymised quotes in research materials.

## Results

### Data sharing in MalariaGEN research: developing a data sharing policy

In line with policy changes in the field of genomics more widely [2], MalariaGEN researchers were required to make their data publicly available for secondary use. And, as is now common in genomics, the public release of data was a condition of funding [29]. This required MalariaGEN researchers to develop mechanisms and policies for data release.

The MalariaGEN researchers developed a data sharing policy in a number of distinct stages. First, researchers developed a discussion paper describing the different kinds of clinical and genomic data generated by the collaboration, and various options for regulating data access. This discussion paper was circulated to funders, MalariaGEN principal investigators, and ethics committees in Africa, Asia and Europe that had approved the MalariaGEN study. Comments from all of these stakeholders were considered in determining the most appropriate way to share the MalariaGEN genomic data. The subsequent data release policy was again circulated to the various stakeholders for input and finalisation.

Two important ethical concerns about data sharing practice emerged during the development of the draft MalariaGEN data release policy. The first of these was a concern that the unmediated sharing of data might have the potential to disproportionately benefit researchers outside of Africa, who had not contributed to sample or data collection but who had the means to analyse vast amounts of data much more quickly than those in low-income countries who had played a key role in producing it. We have discussed this aspect of MalariaGEN’s approach to data sharing elsewhere [5]. The second was a concern about whether MalariaGEN data might have the potential to be used in ways which could harm ethnic groups and what might be done to minimise the risk of this. In this paper we will focus on the second of these issues.

Whilst there had been some discussion of the implications of ethnicity data earlier in the life of MalariaGEN, concerns about the implications of using ethnicity data became more prominent in discussions about data sharing. Indeed, the very first document prepared by the MalariaGEN researchers to discuss data sharing identified the possibility of ‘ethnic stigmatisation’ – a concern that had not been discussed by the researchers before. The data release discussion paper, which was prepared to describe the kinds of data that were generated and to introduce the topic of data sharing, read that

“any information on ethnic group, geographical location or country linked to individual-level genetic data could potentially provide a wealth of information about the people in that ethnic group, location or country.[…] the greater the detail of ethnic or other demographic data that is linked to the genetic data released, the greater the need to protect the ethnic groups or communities involved, potentially through restrictions on the use of the data” (Data Release Discussion Paper, 2006).

This discussion document was circulated to a wide range of people with an interest in the MalariaGEN project, including the funding bodies and members of ethics committees that had reviewed and approved MalariaGEN studies. In response to this, one of the two MalariaGEN funding bodies also explicitly questioned the potential that MalariaGEN genomic data could be used to harm African population groups. For instance, one member of a MalariaGEN funding body queried

“are you comfortable that there is no risk in releasing the open access data to all? I.e. [is there] no risk of stigmatisation?” (Funding Body Representative email to MalariaGEN, 2006).

Such concerns had not been articulated or expressed in the collaboration before discussions about data release. What became clear in these discussions is that MalariaGEN researchers considered themselves under an obligation to protect individuals and communities, and deliberated whether it would be possible and desirable to extend this obligation to other data users.

“it may be appropriate to pass on an obligation to the data users to only use the data for the purposes of studying candidate genes in malaria resistance and not to use the data in any way that may lead to ethnic stigmatisation” (Data Release Discussion Paper, 2006).

The researchers in the collaboration tried at this stage to determine the best possible mechanism for data release. This included consideration of both a completely open access option where information about ethnic groups was coded, and a managed or restricted access option. Our analysis showed that the ultimate decision by MalariaGEN to adopt a ‘managed’ approach to data access originated from a desire to exclude the possibility of harm to ethnic groups that might have resulted from the unrestricted release of genomic data. And although various options for the release of genomics data were discussed, researchers came to the view that data should not be released for secondary research use without prior vetting of secondary users and their proposed projects.

MalariaGEN researchers largely described themselves as being strongly in favour of open access policies genomic science, in the expectation that this would improve the utility of data and lead to greater innovation.

“it makes the science move a lot faster and I also think it is a good thing because otherwise the very few very well funded labs basically get to dominate the scientific discourse…” (R).

Researchers recognised that managing data access countered the prevailing norms and funders’ expectations, and that it would be important for them to be able to provide a very clear and strong justification for placing any restrictions on the release of data.

“including ethnic groups in the dataset is a concern but [the Consortium] need(s) to be able to justify any reduction in the level of information we release” (Minutes of the 15th MalariaGEN Programme Management Committee, 2006).

When asked for feedback on the proposed data release policy, one MalariaGEN researcher commented that

“it’s extremely difficult to judge the right point between excessive release of data and undue concealment from the international community” (PI Comments on Second draft Data Release Policy, 2006).

In the end, after much deliberation, on balance, researchers came to the view that their ‘obligation to protect’ meant that arguments in favour of a more managed approach to data release outweighed those in favour of ‘open access’ , and the decision was made to only release data after review of proposals for secondary analysis by a dedicated, independent data access committee.

### Data sharing and changes in practice

On first impression, the decision to manage data access for secondary use seemed puzzling – particularly as it was made by a group of researchers who were vocally committed to the open access agenda. Were this commitment not evident, it might have been tempting to draw the conclusion that concerns about ethnic stigmatisation had simply provided the researchers with a reason to restrict access to project data. But this explanation was not credible given the strength of the researchers’ commitment to open access. Against this background, we were interested to investigate the factors explaining why concern over possible harm to ethnic groups came to emerge in the context of data sharing discussions in MalariaGEN and how it came to influence the development of the model of managed data release adopted. The interviews we conducted were aimed at unraveling the reasons that could explain our observations. In the remainder of this paper, we will discuss why MalariaGEN researchers consider the risk of harm to ethnic groups to be more pronounced when data are analyzed by secondary researchers. We identified three aspects of data sharing policies that researchers considered problematic.

### Data release and the relation to consent and ethics approval

For MalariaGEN researchers, the requirement to share data for secondary analysis of any kind was perceived to be at least potentially incompatible with some aspects of the specific consent that they obtained for their studies.

“the consent was that we were going to work on malaria. So if it just turned into trying to find out [other things] that is sort of betraying the confidence that people give us to do this” (R).

Challenges to obtaining informed consent for research in low-income countries have been well-documented [30-32]. Many of these same challenges are also important when seeking consent to genomics research [33] and interviewees in our project recognised these. Although consent remains of crucial importance in ensuring that genomic research is ethical, we have previously made an argument that there is a need for additional safeguards to protect participants in genomics research because of challenges in obtaining appropriate informed consent [5,33]. The researchers we interviewed in our study seemed to reiterate this point. For instance,

“[you have] to be scrupulously honest to yourself as an investigator and as a group of investigators with the trust of the community and respecting the communities, the things that people have entrusted you to do. I think that is where we tend to operate rather than going to every level of information at the consent level” (R).

Currently when data is shared through public databases, they are shared according to the text that was written in the consent documentation – for instance, if this identified a particular cluster of diseases such as ‘infectious disease’ , then data ought only be shared for such research. However, what this does not do is acknowledge that some participants may not have understood the documentation in this detail and that consent may have been given on the basis of trust. In addition to consent itself, the relation between ethics approval and secondary use was also identified as potentially problematic by interviewees. First of all, ethics approval tends to be granted for a specific piece of work, but when data are shared it can be used for many different types of projects.

“Now a proposal received ethical review based on what was presented […] now to look at these other new areas being uncovered would not be ethical because approval is given based on what was presented” (R12).

The challenge is not the sharing of genomic data per se, but the absence of any scrutiny of secondary research questions.

“we have nothing against getting data in a central library. But what we are against is that unauthorized usage of that data” (REC).

### Understanding the intentions and values of unknown secondary data users

The interviewees also identified the perceived anonymity of the secondary data user, who is unknown to the primary researchers and the ethics committees that approved data release, as a potential problem. Within the MalariaGEN collaboration, researchers know each other, share a commitment to use genomic data to investigate a particular disease and may even, despite their obvious diversity in many respects, see themselves as sharing important relevant values.

“we know all the PIs, all the people working within MalariaGEN and we have signed an agreement […] and mostly we know them in term of their ethical you know… And we know the probability of [abuse] is very low […]. But outside us we don’t know anything about people” (R).

Secondary users are often unknown to the primary researchers, and there is no way to assess their values and past behaviour. In addition, it is difficult to hold secondary users accountable.

“okay because somebody can sit in a sea in a boat, can play with the data and can just [write] something very bad about one ethnic group by using our data, by a bad intention. It is just to avoid the bad intention because… we are thinking about health and participant protection, but somebody else won’t care about that” (R).

There is, moreover, no possibility to investigate the intentions of secondary researchers regarding re-analysis: no scrutiny by ethics committees and no formalisation of ethical obligations.

“The problem arises with what I want to call second degree research. Because those are relying on […] the Internet to make extrapolations and make their own interpretations […]. That is a difficult person to catch in terms of ethics because he will be doing research from secondary materials. […] That is a difficult person to catch and that is a big ethical challenge” (REC1).

Another challenge identified by respondents is that secondary data analysis does not require the formulation of a hypothesis. Datasets can be mined for a wide range of purposes and there is no incentive for secondary data users to use data in accordance with the purpose for which data was collected.

“those who collect they stay true to what they are doing but it’s when it becomes available and then people ask different questions and things which were not intended or probably not even thought about by those who designed the original studies that’s where the problems generally arise” (R).

And datasets are not released in isolation; rather, it is possible that other, complementary datasets are available that could be combined with the MalariaGEN datasets to create a much richer source of information about ethnic groups. This was also a concern for some researchers.

“if there are people who are conducting work in that area and they compare […] sequence data from those sorts of studies and find those traits present in particular sub-groups of our population […] and write a paper independently of anything to do with us because our information might be made publicly available then they could misinterpret or cause offence or problems amongst sub-groups of the population […]

I: is that likely with this kind of data?

It depends, it all depends on what phenotypic and other sort of data go along with it, how possible it is for people to join up datasets” (R).

In literature, this has been referred to as the ‘data environment’ [34]. What our research revealed is that for MalariaGEN researchers, the commitment to sharing genomic data widely for a wide range of purposes raises ethical concerns about possible harms that might arise from secondary use.

### Understanding the context of sample collection

A third and related ethical challenge in data sharing identified by respondents concerned the implications of the absence of any accompanying contextual knowledge, what we would like to call ‘ethical metadata’ , when genomic data are released. In data sharing, it is considered important that the datasets are ‘anonymous’: they are treated as isolated and unlinked collections of genomic data that can be transferred without any information on research participants, their populations and the location and time of sample collection. When genomic data are shared, there is no transfer of ‘embedded’ knowledge about groups, the original research project and questions, local relations and sensitivities. The researchers we interviewed considered this separation of the data from important contextual information potentially problematic.

“[The local scientist] understands better the cultural background of the project and the communities and all these kind of things. And the local scientist has responsibility of considering the consequences of the science that he does” (R).

MalariaGEN researchers collect samples in many rural areas in Africa, and research participants are generally characterized by relatively low income and education levels [35]. Possibly because of this, many of the researchers involved in the collaboration strongly perceived themselves to have an obligation to protect research participants from harm.

“these type of studies are profoundly embedded in a culture, in a scientific attitude that is not [participants’] culture. […] In a sense we’re asking a lot from them you know it’s a sort of carte blanche yes. So you know ‘we don’t understand what you want us to do but okay we trust [you]’” (R4).

For researchers, knowledge of the contextual features of this ‘original’ trust relationship between research participants and the research team – such as for example, the person who obtained consent - was essential in ensuring that genomic data were analyzed appropriately. In addition, understanding the relationships between ethnic groups in the setting was seen to be important to ensure that genomics research findings would be reported appropriately. Where relationships between ethnic groups are strained, for instance, it is more likely that those who knew about this and understood its importance would take care when reporting on genetic relatedness of groups.

Within the boundaries of the MalariaGEN research collaboration, researchers felt confident that their insider knowledge and values were appropriately articulated, shared and respected.

“if I’m working in my small area then I know I’m responsible for the status there and I have to keep it this way…. when I agree to a big project like this then a lot of things are going out of my hands but again the same trust that the community posed in me, I’m expecting that trust in this bigger project” (R).

But they were concerned that outside of the boundaries and hence the shared values and practices of the MalariaGEN consortium, there was not the same degree of certainty that these values would be understood or respected.

## Discussion

### Mechanisms preventing harm in genomics research

Taken together, what interviewees seemed to be saying was that when genomic datasets are shared with secondary users, they are generally shared as objects isolated from important – and protective – normative context and this raises particular ethical issues. Interviewees described three problematic aspects of data sharing arising out of this isolation, namely the relation to informed consent and ethics approval; understanding the intentions and values of unknown secondary data users; and understanding the cultural background of sample donors. Isolation from these aspects is important because they form a ‘shared normative culture’ consisting of trust relationships, a shared morality and in-depth understanding of the context of sample collection.

A first, and often largely implicit, component of the normative culture in MalariaGEN is trust. Interviewees identified trust relationships between participants and their research team; between MalariaGEN researchers in different settings; and between researchers and ethics committees. Trust between researchers included a shared, mutual understanding that data will not be used in ways that could harm research participants. The consequence of violating this trust would likely be an end to (future) collaboration – an important consequence in the context of genomics research where collaboration is essential for successful research. Trust between participants and the research team was described as important by fieldworkers and researchers, and relates to a shared obligation to protect research participants. In literature, this perceived obligation has been termed ‘custodianship’ [8], which signifies that researchers often perceive themselves to be custodians of data or samples, on behalf of communities or individuals that donated these. Trust was also identified as an important aspect of the research process by research ethics committee members. In that case, the questions of trust related to REC members’ expectation that researchers would respect the terms and limitations of the ethics approval for their project. The fact that researchers and ethics committee members were often members of the same institution was identified as significant in this respect. Even in the absence of formal auditing mechanisms, it was seen as vitally important for researchers to preserve their good standing at their own institution, and to remain known as a person of good moral standing.

A second component of the normative culture in MalariaGEN is what we have chosen to call a shared morality between researchers [36]. This has much in common with the role of trust because the possibility of trust is at least in part based on a perceived mutual understanding of appropriate, ethical behaviour and of the obligations of researchers to participants, to each other and to research ethics committees. The respondents for this project shared views on important ethical issues such as maintaining confidentiality and appropriate re-use of data. Importantly, researchers also shared the ambition to investigate a disease that is detrimental to the wellbeing of many people in the developing world. Together, the shared values and desire to increase knowledge about a detrimental disease seems to constitute the core of a shared morality. This shared morality is largely implicit, but is also, on occasion, made explicit for instance in the contracts drawn between the various institutions in MalariaGEN or in the development of shared policies such as those on data sharing or consent.

A third aspect of the normative culture in MalariaGEN is detailed knowledge of the context of sample collection – or the fact that there is always at least one person in the collaboration who might be consulted about this. Interviewees identified two types of contextual knowledge to be important. The first of these was knowledge of the (limitations of) consent and ethics approval given for the study. Knowing what was approved, and having a reasonable understanding of participant expectations were identified as important in preventing inappropriate research questions being investigated. The important thing about this knowledge is that it was always knowledge which included but went beyond what was included in the form itself. The second type of contextual knowledge identified by the interviewees relates to detailed knowledge of the relations between, and customs and traditions of, the ethnic groups included in the study. According to the interviewees, such knowledge is essential in assessing the potential for research findings to cause harm to ethnic groups and was a key factor informing the development of appropriate and sensitive practices.

Together, these three components of the normative culture in MalariaGEN are seen by our interviewees to provide a safeguard against harm. When data were to be shared with people outside of the original collaboration, researchers could no longer trust in the ethical common ground to prevent harmful uses of data – calling into being concerns about ethnic stigmatisation. In order to address such concerns, it may be necessary to accompany genomic data with relevant information about the normative context of research. This could include for instance information about the informed consent process and the culture of and relations between ethnic groups. We would like to call this information about ethical aspects of research ‘ethical metadata’. Just as metadata provides information about the scientific processes that led to data, ethical metadata would also provide information about ethical aspects of genomic data. In this sense, we believe our research echoes a call for greater ‘ethical reproducibility’ in biomedical research [37].

## Conclusion

Data sharing is now the norm in genomics research. The requirement for data sharing had profound implications for the relationships and values within the MalariaGEN research culture and required researchers to engage critically both with their own commitment to data sharing and their sense of responsibility to research communities. Ethical concerns over the use of ethnic data only emerged in the context of discussions aimed at developing and putting in place a policy for the wider release of data to the international scientific community. In our investigation of why this was the case, we identified a number of problematic aspects of data sharing practices, namely limited ability to record and share information about informed consent and ethics approval, difficulties in assessing the intentions and integrity of secondary data users, and concerns that secondary users could involuntarily inflict reputational damage to population groups by not being knowledgeable of the cultural background of and relations between ethnic groups. Together these aspects are part of a shared normative culture that is less mobile than the data to which it refers. Within MalariaGEN, this normative culture prevents against harm, but is not easily shared together with data.

The data presented in this paper were collected in the context of a wider project that aimed to develop a better understanding of the ethical issues raised by the use of ethnic data in a particular genomics research in Africa. We only interviewed scientists involved with this particular project, many of whom were clinicians primarily and not genomic scientists. We recognise that clinicians may experience a greater burden of care towards research participants than people who simply see and analyse genomic findings. Whether and how, therefore, our results are relevant to other scientists contributing to genomics research projects needs further investigation.

By way of solution we would propose that at least some information about the normative context of sample collection and data sharing – what we called ethical metadata – needs to be taken into account when data sharing decisions are to be made. This may particularly be the case where research is conducted on identifiable population groups where stigma or discrimination are of concern. Where there is concern that data could harm population groups, it is our view that, at a minimum, such data needs to be shared with a description of the relevant features of the context within which data was collected, and to which research results pertain. In addition to information about the consent process, this would include information on ethics approval, and a description of the population groups involved in the research.

## Competing interests

The authors declare that they have no competing interests.

## Authors’ contributions

JdV, MP, RF and DPK conceived the project. JdV collected data and drafted the manuscript. JdV, MP and RF analysed the data. TNW, KB and DPK assisted in data collection and analysis. MP, RF, TNW, KB and DPK provided critical feedback on early drafts of the manuscript. All authors read and approved the final manuscript.

## Pre-publication history

The pre-publication history for this paper can be accessed here:

http://www.biomedcentral.com/1472-6939/15/62/prepub
